# Preclinical Development of Antisense Oligonucleotides to Rescue Aberrant Splicing Caused by an Ultrarare *ABCA4* Variant in a Child with Early-Onset Stargardt Disease

**DOI:** 10.3390/cells13070601

**Published:** 2024-03-29

**Authors:** Nuria Suárez-Herrera, Catherina H. Z. Li, Nico Leijsten, Dyah W. Karjosukarso, Zelia Corradi, Femke Bukkems, Lonneke Duijkers, Frans P. M. Cremers, Carel B. Hoyng, Alejandro Garanto, Rob W. J. Collin

**Affiliations:** 1 Department of Human Genetics, Radboud University Medical Center, 6525 GA Nijmegen, The Netherlands; nuria.suarezherrera@radboudumc.nl (N.S.-H.); nico.leijsten@radboudumc.nl (N.L.); dyah.karjosukarso@radboudumc.nl (D.W.K.); zelia.corradi@radboudumc.nl (Z.C.); femke.bukkems@radboudumc.nl (F.B.); lonneke.duijkers@radboudumc.nl (L.D.); frans.cremers@radboudumc.nl (F.P.M.C.); alex.garanto@radboudumc.nl (A.G.); 2 Department of Ophthalmology, Radboud University Medical Center, 6525 GA Nijmegen, The Netherlands; catherina.li@radboudumc.nl (C.H.Z.L.); carel.hoyng@radboudumc.nl (C.B.H.); 3 Dutch Center for RNA Therapeutics, 2311 EZ Leiden, The Netherlands; 4 Department of Pediatrics, Amalia Children’s Hospital, Radboud University Medical Center, 6525 GA Nijmegen, The Netherlands

**Keywords:** N-of-1, antisense oligonucleotide, splicing modulation, pseudoexon, RNA therapy, *ABCA4*, Stargardt disease

## Abstract

Precision medicine is rapidly gaining recognition in the field of (ultra)rare conditions, where only a few individuals in the world are affected. Clinical trial design for a small number of patients is extremely challenging, and for this reason, the development of N-of-1 strategies is explored to accelerate customized therapy design for rare cases. A strong candidate for this approach is Stargardt disease (STGD1), an autosomal recessive macular degeneration characterized by high genetic and phenotypic heterogeneity. STGD1 is caused by pathogenic variants in *ABCA4*, and amongst them, several deep-intronic variants alter the pre-mRNA splicing process, generally resulting in the insertion of pseudoexons (PEs) into the final transcript. In this study, we describe a 10-year-old girl harboring the unique deep-intronic *ABCA4* variant c.6817-713A>G. Clinically, she presents with typical early-onset STGD1 with a high disease symmetry between her two eyes. Molecularly, we designed antisense oligonucleotides (AONs) to block the produced PE insertion. Splicing rescue was assessed in three different in vitro models: HEK293T cells, fibroblasts, and photoreceptor precursor cells, the last two being derived from the patient. Overall, our research is intended to serve as the basis for a personalized N-of-1 AON-based treatment to stop early vision loss in this patient.

## 1. Introduction

The field of personalized therapy is constantly growing due to the increasing yet unmet demand for treatment of (ultra)rare diseases, especially when only a few patients worldwide are affected or carry a given genetic defect. This interest is further fueled by the improvement of next-generation sequencing platforms to screen patient cohorts. High-throughput approaches revealed a whole set of considerably heterogeneous pathological mechanisms in recent years, meaning that the genetic origin and/or disease progression may differ extremely between individuals with the same diagnosis. As a consequence, the observed heterogeneity may lead to variable responses to treatment, which is a challenging aspect in clinical trials. N-of-1 strategies have been developed over time to speed up tailor-made therapy design for smaller patient groups [1,2]. The main goal for this type of intervention is to properly assess whether it brings significant benefit to the target individual. Depending on the case, N-of-1 clinical trial design can vary from a basic single case design following interrupted time series to a reversal design where intervention and interruption periods are repeated, with or without intermediate washout periods. In all these cases, it is of high importance to consider the outcome measures to demonstrate individual responses. Ultimately, and even though N-of-1 trials are focused on a single individual, the collected data from each specific case should be aggregated to improve future interventions [3].

Both allelic and symptomatic heterogeneity is a characteristic aspect of *ABCA4*-associated Stargardt disease (STGD1, OMIM: 248200). STGD1 is the most common type of Mendelian (or inherited) macular dystrophy and falls within the category of inherited retinal diseases (IRDs), a large group of clinically and genetically heterogeneous and rare conditions affecting the retina, which can eventually lead to blindness [4]. Biallelic variants in *ABCA4* can cause STGD1, and currently, more than 2400 unique variants have been found within the locus (www.lovd.nl/ABCA4, last accessed on 12 July 2023). The *ABCA4* gene encodes the ATP-binding cassette A4 (ABCA4) protein, a flippase localized at the rim of rod photoreceptor disc membranes and throughout the cone photoreceptor cell membrane. Impairment of its activity due to the presence of pathogenic variants leads to the accumulation of toxic derivatives in the retina and causes progressive degeneration of photoreceptor cells [5,6]. Coding variants represent most of the variants in *ABCA4*. However, there is a significant proportion of mutations affecting the pre-mRNA splicing process, including many deep-intronic *ABCA4* variants [7]. These represent approximately 10% of the reported alleles in recent patient cohort screenings [8], and they can range from a relatively high frequency (e.g., c.4253+43G>A, c.4539+2001G>A, c.5196+1137G>A, c.4539+2064C>T and c.769-784C>T) to ultrarare, meaning that only one or a few patients worldwide are harboring these variants. Therefore, STGD1 patients are potential candidates for therapy development following N-of-1 clinical trial designs.

Generally, deep-intronic variants promote the recognition of cryptic splice sites within the intronic regions of the gene. As a consequence, they lead to the inclusion of pseudoexons (PEs) into the final transcript, often resulting in premature termination of protein synthesis. This type of splicing alteration is also true for variant c.6817-713A>G, located in the last intron of *ABCA4*, that was discovered as one of the two pathogenic *ABCA4* alleles in a single STGD1 case [9].

A common strategy to tackle these splicing alterations and restore correct transcript employs antisense oligonucleotides (AONs). AONs are short and chemically modified single-stranded RNAs that can modulate splicing by base pairing to the target pre-mRNA sequence and interfering with the spliceosome binding [10]. Previously described intronic variants in *ABCA4* have been targeted with this strategy, and the corresponding splicing defects were successfully rescued in vitro [11,12,13,14,15,16]. Despite the extended applicability of AONs to correct different splicing alterations, the existence of rare and ultrarare cases limits their initial evaluation in a clinical setting. Therefore, customized assessment of these molecules by implementing N-of-1 designs might be the prospective means of accelerating clinical intervention in STGD1 patients. An increasing number of examples in the literature are encouraging the implementation of these clinical trial designs in a broader number of diseases [17,18,19,20,21]. More concretely with respect to the use of AONs, milasen is the chief example of N-of-1 implementation [22]. The impressive fastness in clinical intervention helped increase the quality of life of the affected child, and despite its inability to reverse the disease’s late-stage-associated symptoms, it illustrates how valuable N-of-1 interventions can be when developing therapeutic options for ultrarare cases. Additionally, recent guidelines on AON design from an N-of-1 perspective are available to combine the efforts of preclinical researchers and clinicians in order to maintain adequate scientific standards [23].

Altogether, these outcomes inspired this research to be focused on a specific case of an early-onset STGD1 carrying the ultrarare *ABCA4* variant c.6817-713A>G. We aimed to rescue the resulting splicing defect by designing several AON candidates and testing them in three different cell models: HEK293T cells, fibroblasts, and photoreceptor precursor cells (PPCs). The last two models were derived from the affected individual in order to adequately assess the effect of the different tested conditions. Ultimately, our research aims to contribute to the development of clinical intervention, applying the N-of-1 design recommendations and being, to our knowledge, the first instance of its implementation for a specific STGD1 case.

## 2. Materials and Methods

### 2.1. Clinical Evaluation

Clinical data of the patient were collected from her medical records. The parents of the underaged patient were informed of the procedures and provided informed consent for additional imaging and skin biopsy. Best-corrected visual acuity was assessed using an ETDRS grid. Both eyes were examined through dilated slit lamp examination and fundoscopy. Fundus autofluorescence imaging and optical coherence tomography (OCT) scans were acquired using Spectralis (Spectralis, Heidelberg Engineering, Heidelberg, Germany). Color fundus photographs were taken using the Topcon TRC50IX camera (Topcon Corporation, Tokyo, Japan).

### 2.2. Antisense Oligonucleotide Design

The four AONs used in this study were designed according to previously described guidelines [24,25]. The accessibility of the target RNA secondary structure was analyzed by Mfold software version 6.4 [26]. Splicing enhancer motifs SC35 were predicted with ESE finder [27], whereas sequence properties such as the length, GC content, and Tm of the AONs were calculated using the OligoCalc online software version 3.27 [28], all reported in Table S1. Further details on the region employed for AON design are described in Figure S1. Customized oligonucleotides were purchased from Eurogentec (Liege, Belgium), all containing the phosphorothioate (PS) backbone and 2′-*O*-methoxy-ethyl (2′-MOE) sugar ring chemical modifications, including the scrambled oligonucleotide (SON). All lyophilized AONs and SON were resuspended in sterile PBS to a working concentration of 100 µM.

### 2.3. Generation of Wild-Type and c.6817-713A>G ABCA4 Splice Vectors

The wild-type and mutant minigenes were previously generated by cloning a genomic *ABCA4* region into the Gateway^®^ pCI-NEO-*RHO* destination vector (Thermo Fisher Scientific, Waltham, MA, USA). The resulting constructs harbor a part of intron 49 (g.94,460,003_g.94,459,176, GRCh37/hg19 genomic positions) [9].

### 2.4. Cloning of PE in ABCA4 cDNA Vector

To check the effect of the PE insertion at the protein level, the entry clone pDONR201 harboring the *ABCA4* cDNA without start codon, kindly provided by Prof. R.S. Molday, was used for site-directed insertion. Primers (Table S2) were designed to achieve the insertion of the first 33 nt of the PE (c.6817-835_c.6817-803) between exons 49 and 50 of *ABCA4* cDNA (until and including the premature termination codon (PTC), predicted with ORF finder, NCBI), both primers contain a complementary arm to the template and a non-complementary arm with the additional PE sequence (overlap of 10 nt between non-complementary arms). Reaction mixture (50 µL) contained 0.5 µM of each primer, 0.2 mM dNTPs, 1 U Phusion High-Fidelity DNA Polymerase (New England Biolabs, Leiden, The Netherlands), 1X Phusion HF Buffer, 0.5X Q-solution (Qiagen, Venlo, The Netherlands), and 20 ng plasmid as a template. PCR conditions were initial denaturation at 98 °C for 30 s, 15 cycles at 98 °C for 10 s each, annealing 62 °C and extension at 72 °C (1 min/kb), with a final extension at 72 °C for 10 min. PCR products were incubated with DpnI (New England Biolabs, Leiden, The Netherlands) for 4.5 h at 37 °C, followed by heat inactivation at 80 °C for 20 min. The obtained clones were validated by restriction analysis and Sanger sequencing to validate the presence of the insertion (sequencing primers listed in Table S3). Last, the validated entry clone was cloned into the Gateway^®^ p3xHA_CMV/DEST destination vector following the afore-mentioned instructions.

### 2.5. In Vitro AON Rescue Studies in HEK293T Cells

Human embryonic kidney (HEK293T, ATCC# CRL-3216™) cells were cultured in DMEM supplemented with 10% fetal bovine serum (FBS), 1% penicillin-streptomycin, and 1% sodium pyruvate at 37 °C and 5% CO_2_. To test the designed AONs, HEK293T cells were seeded in a six-well plate at a 70% confluence. Four hours post-seeding, cells were transfected with 1 µg of either the wild-type or c.6817-713A>G *ABCA4* minigene or left non-transfected as endogenous control. The next day, cells were detached with trypsine and split into 5 wells of a 12-well plate. After four hours, the corresponding AONs or SON were transfected at a final concentration of 0.5 µM, and at least one well was kept non-treated in parallel. Minigene transfections and oligonucleotide delivery were performed with FuGENE HD reagent (Promega, Madison, WI, USA) as described in previous protocols [24,29]. After 48 h, cells were harvested for further RNA analysis. Experiments were performed in three independent replicates.

### 2.6. ABCA4 cDNA Vector Transfection in HEK293T Cells

To evaluate the PE effect at protein level, HEK293T cells were seeded in a six-well plate at a 70% confluence. Four hours post-seeding, cells were transfected with 1 µg of either the wild-type or 33-bp insertion *ABCA4* cDNA construct or left untransfected as endogenous control. Construct transfection was performed with FuGENE HD reagent (Promega, Madison, WI, USA) as mentioned in the previous section. Forty-eight hours post-transfection, cells were harvested for further protein analysis.

### 2.7. AON Rescue Studies in Patient-Derived Fibroblasts

Fibroblast cell line was derived from the affected patient and a healthy control individual. Patient carried c.6817-713A>G (p.[=,Gln2272_Asp2273ins*11]) on allele 1 and c.3259G>A (p.(Glu1087Lys)) on allele 2, and healthy control carried the wild-type *ABCA4* gene. Fibroblasts maintenance was conducted in DMEM supplemented with 20% FBS, 1% penicillin--streptomycin, and 1% sodium pyruvate at 37 °C and 5% CO_2_. For AON testing, 350,000 cells/well were seeded in six-well plates. After overnight incubation, fibroblasts were transfected with the corresponding AON or SON at 0.1, 0.25, and 0.5 µM concentrations in 1 mL of medium or left non-treated. After six hours, an additional 1 mL of the medium was added to all wells. Transfection protocol was followed as described elsewhere [24]. Forty-eight hours after AON delivery, fibroblasts were harvested for further RNA analysis. All experiments were performed in three independent replicates.

### 2.8. Patient-Derived iPSC Culture Conditions

Lentiviral transduction was employed to reprogram patient-derived fibroblasts into iPSCs (RMCGENi021-A, https://hpscreg.eu, last update on 21 December 2023). The control iPSC line was also reprogrammed via lentiviral transduction from an age- and gender-matched healthy individual. The lentiviral particles were obtained from pRRL_PPT_SF_hOct34co_hKlf4co_hSox2co_hmyc_idTomato_pre_FRT plasmid [30]. Afterwards, the cells were then seeded on inactivated mouse embryonic fibroblasts from which colonies were picked and expanded by the Stem Cell Technology Center (Radboudumc, Nijmegen, The Netherlands). iPSCs were cultured at 37 °C and 5% CO_2_ in Essential 8™ Flex Medium (E8F, Gibco, Fort Worth, TX, USA) supplemented with 100 µg/mL Primocin^®^ (InvivoGen, Toulouse, France). Maintenance of iPSC lines was conducted on six-well plates coated with Matrigel^®^ Growth Factor Reduced (GFR) Basement Membrane Matrix (Corning, Corning, NY, USA) at 1:50 dilution in DMEM/F12.

### 2.9. iPSC Differentiation to PPCs

To derive PPCs from iPSCs, a 30-day differentiation protocol was adapted from previous research on 2D to 3D retinal organoid differentiation [31,32]. Cell seeding was conducted in clumps at 1:10 dilution by gently detaching the iPSC culture with ReLesR solution (Stemcell Technologies, Zaandam, The Netherlands), transferring them into 12-well plates previously coated with Matrigel^®^ at 1:20 dilution and containing E8F supplemented with RevitaCell (Gibco, TX, USA). The next day, E8F medium was completely refreshed without RevitaCell supplement, and iPSC were cultured as mentioned until reaching full confluency. On day 0, medium was replaced with 2 mL of Essential 6™ medium (Gibco, TX, USA) and refreshed the next day. On day 2, medium was changed to 2 mL of neural induction medium (NIM), which consisted of Advanced DMEM/F12 medium (Gibco, TX, USA) supplemented with N-2 (Gibco, TX, USA), GlutaMAX solution (Gibco, TX, USA) and 100 µg/mL Primocin (InvivoGen, Toulouse, France). NIM was completely replaced again on day 4, and on day 6, NIM was again refreshed but supplemented with 1.5 nM BMP4 (Sigma-Aldrich, St. Louis, MO, USA). BMP4 was resuspended in 5 mM HCl to a final concentration of 1.5 µM. From this day on, half of the medium in each well was replaced three times a week until day 30.

### 2.10. AON Rescue Studies in Patient-Derived PPCs

On day 20, AONs were delivered at three different concentrations (0.25, 0.5, and 1 µM), whereas the SON was delivered only at the highest concentration (1 µM). Oligonucleotides were gymnotically delivered in 750 µL of NIM to each well (without using a delivery agent). In the case of RNA analyses, cycloheximide (CHX, Sigma-Aldrich, St. Louis, MO, USA) was added to the medium at 100 µg/mL final concentration to inhibit nonsense-mediated decay (NMD) and incubated for the next 24 h. For both control and patient lines, one of the wells was kept as non-treated (NT). CHX is used to visualize the accumulation of aberrant transcripts in those cell lines with active NMD (such as the case of PPCs), which degrades transcripts containing PTCs [33], usually present within the sequence of PEs or as a cause of the change in the reading frame. For protein analyses, CHX treatment was not performed since it inhibits protein translation. On day 30, cells were washed with PBS and harvested for further RNA and protein analysis. All differentiation experiments were performed in two independent replicates.

### 2.11. RNA Isolation and cDNA Synthesis

RNA was isolated from HEK293T cells, fibroblasts, and PPCs using the Nucleospin RNA kit (Macherey-Nagel, Düren, Nordrhein-Westfalen, Germany) as indicated by the manufacturer. For HEK293T cell samples, 1 µg of total RNA was used as initial input for the cDNA synthesis reaction, which was performed with iScript cDNA Synthesis kit (Bio-Rad, Lunteren, The Netherlands) to a final concentration of 50 ng/µL. For fibroblast and PPC samples, RNA was first precipitated with ethanol as indicated in previous protocols [34]. Afterwards, cDNA synthesis (20 µL) was performed with 1 µg of total RNA as described in the SuperScript VILO Master Mix (Thermo Fisher Scientific, Waltham, MA, USA) kit’s protocol.

### 2.12. PPC Characterization by qPCR

For differentiation markers analyses, 12.5 ng of template cDNA from either the corresponding iPSC (Day 0) and PPC (Day 30) lines were used for quantitative PCR. Reactions were prepared with GoTaq Real-Time Quantitative PCR Master kit (Promega, WI, USA), following manufacturer’s recommendations, and performed in Applied Biosystem QuantStudio 5 Digital system. Expression levels of nine PPC markers and one housekeeping gene (*GUSB*) were analyzed in order to evaluate the differentiation of the two iPSC lines into PPCs. Primers for qPCR markers detection are listed in Table S4. Expression of *GUSB* housekeeping gene was used to normalize each sample, and relative expression levels from each marker in PPCs (Day 30) were compared to the respective iPSCs (Day 0) using the 2^−ΔΔCt^ method [35].

### 2.13. iPSC Characterization

The patient-derived iPSC line was maintained as previously mentioned. The characterization experiments were performed with cells at passage 15–20. For immunocytochemistry studies, iPSCs were fixed in 2% paraformaldehyde in PBS for 20 min, followed by permeabilization in 1% Triton X-100 for 5 min. After 20 min blocking with 2% BSA in PBS, the cells were incubated for 2 h at RT with the following primary antibodies: rabbit anti-OCT4 (1:200 dilution, Abcam, Cambridge, UK), rabbit anti-NANOG (1:100 dilution, Abcam, Cambridge, UK), and mouse anti-SSEA4 (1:100 dilution, Abcam, Cambridge, UK), then washed three times with PBS. Afterwards, iPSCs were incubated for 1 h at RT with the corresponding secondary antibody solutions: goat anti-rabbit 568 (1:500 dilution, Thermo Fisher, Waltham, MA, USA) and goat anti-mouse 488 (1:500 dilution, Thermo Fisher, Waltham, MA, USA). Cells were mounted with Vectashield with DAPI (Vector Laboratories, Newark, CA, USA). Microscopy images were taken at 20x using Zeiss Axio Imager (scale bar: 100 µm). Trilineage differentiation was performed using Stemdiff Trilineage differentiation kit (Stemcell Technologies, Zaandam, The Netherlands) according to the manufacturer’s instructions. The differentiation was analyzed by qPCR as previously indicated and using primers listed in Table S5. Pluripotency of the patient-derived iPSCs was also analyzed via qPCR using primers listed in Table S5.

Since the patient-derived iPSC line was generated by polycistronic lentiviral reprogramming, the integration and silencing of the reprogramming factors were analyzed via PCR on gDNA (10 ng) and cDNA (25 ng). The three selected sites of the vector were *OCT4/KLF4*, *SOX2/c-myc*, and *dTomato*, and the designed primers for this PCR are listed in Table S5. The plasmid pRRL_PPT_SF_hOct34co_hKlf4co_hSox2co_hmyc_idTomato _pre_FRT [30] was used as positive control. The absence of mycoplasma contamination in the culture was determined with the VenorGEM Classic mycoplasma detection kit (Minerva Biolabs, Berlin, Germany) following the manufacturer’s manual. AmpFLSTR identifier PCR amplification kit (Life Technologies, Bleiswijk, The Netherlands) was employed for STR analysis of 16 loci. CytoScan HD Array Kit (Applied Biosystems, Waltham, MA, USA) was used to search for chromosomal aberrations.

### 2.14. ABCA4 Transcript Analysis

Splicing correction in all cell models was assessed by RT-PCR. In the case of HEK293T splice assays, primers were located in exon 3 (forward) and 5 (reverse) of the *RHO* gene, and amplification of *RHO* exon 5 was used as minigene transfection control. For patient-derived fibroblasts and PPCs, the designed primers were located on *ABCA4* exon 49 (forward) and 3′-UTR (reverse). In all cases, RT-PCR of *ACTB* exons 3 and 4 was used as a loading control. A list of primers for transcript analysis is included in Table S6. For HEK293T assays, all reaction mixtures (25 µL) contained 10 µM of each primer pair, 2 mM of dNTPs, 1.5 mM MgCl_2_, 0.5X Q-solution (Qiagen, Venlo, The Netherlands), 1U of Taq polymerase (Roche, Penzberg, Germany), and 50 ng of input cDNA. PCR conditions were 94 °C for 2 min, followed by 35 cycles of 30 s at 94 °C, 30 s at 58 °C, and 30 s at 72 °C, with a final extension step of 5 min at 72 °C. PCR products were resolved in 2% (*w*/*v*) agarose gel (10 µL for *RHO* exon3-5 PCR product, and 5 µL for *RHO* exon 5 and *ACTB* PCR products). For patient-derived fibroblasts, *ABCA4* PCR mixture contained 100 ng of input cDNA, whereas for patient-derived PPCs, 50 ng of input cDNA were used. In both cases, 50 ng were used for the *ACTB* PCR mixtures as input cDNA. PCR conditions were 94 °C for 2 min, followed by 35 cycles of 30 s at 94 °C, 30 s at 58 °C, and 70 s at 72 °C, with a final extension step of 2 min at 72 °C. All PCR products were resolved on 2% (*w*/*v*) agarose gels (25 or 10 µL for *ABCA4* PCR products from fibroblasts or PPCs, respectively, and 5 µL for *ACTB* PCR products were loaded on gel). All transcripts were validated by Sanger sequencing (Figure S2). Semi-quantitative analysis of both correct and aberrant transcripts was performed with Fiji software version 1.53t (Table S7) [36].

### 2.15. Protein Isolation and Western Blot Analysis

HEK293T cells and PPCs cells were washed with PBS and homogenization was helped by mechanical scrapping in 200 µL of ice-cold RIPA protein lysis buffer (50 mM Tris pH 7.5, 1 mM EDTA, 150 mM NaCl, 0.5% Na-Deoxycholate, 1% NP-40, 0.75% SDS) with cOmplete™ Mini Protease Inhibitor Cocktail (Roche). Protein lysates were incubated on ice for an hour prior to sonication. Protein concentration was quantified using the Pierce™ BCA Protein Assay Kit (Thermo Fisher Scientific, Waltham, MA, USA) according to the manufacturer’s instructions. Protein samples (10 µg for HEK293T cells and 100 µg for patient-derived PPCs) were loaded on 4–15% Mini-PROTEAN^®^ TGX Stain-Free™ pre-cast gels (Bio-Rad, Lunteren, The Netherlands) and ran for 30 min at 250 V in Tris-Glycine-SDS buffer (Bio-Rad, Lunteren, The Netherlands). Transfer was performed on Trans-Blot Turbo Mini 0.2 µm nitrocellulose membranes (Bio-Rad, Lunteren, The Netherlands) for 7 min at 25 V and constant 1.3 A on the Trans-Blot Turbo Transfer System (Bio-Rad, Lunteren, The Netherlands). Membranes were blocked in 5% non-fat milk in PBS for 1 h at RT and incubated overnight at 4 °C with the appropriate primary antibodies diluted in 2.5% non-fat milk in PBS. Rabbit anti-ABCA4 (1:1000 dilution, Abcam, Cambridge, UK), rabbit anti-HA (1:1000 dilution, Sigma-Aldrich, Saint Louis, MO, USA), and rabbit anti-β-tubulin (1:1000 dilution, Abcam, Cambridge, UK) antibodies were used. Blots were washed in PBS-0.1% Tween (3 times, 5 min), incubated with goat anti-rabbit IRDye-800 and goat anti-rabbit IRDye 680 (1:10,000 dilution, Li-COR Biosciences, Lincoln, NE, USA) for 1 h at RT in the dark. Membranes were washed in PBS-0.1% Tween (3 times, 5 min) and developed in the Odyssey Imaging System (Li-COR Biosciences, Lincoln, NE, USA). Intensity of the ABCA4 bands was semi-quantified using Image J software version 1.53t, normalizing each sample to its corresponding β-tubulin and to the non-treated (NT) control or patient PPCs (Table S8).

### 2.16. Statistical Analysis

Data were expressed as means ± SD and processed with GraphPad Prism 9 software (GraphPad, San Diego, CA, USA). To analyze the differences between the tested conditions, we employed the one-way ANOVA test with subsequent Dunnett’s multiple comparison analysis, having the non-transfected or the CHX-treated column as reference for correct transcript levels for HEK293T cells and fibroblasts, or PPC studies, respectively. Statistical significance was determined for *p*-values smaller than 0.05, presented with one or multiple asterisks (* *p* < 0.05, ** *p* < 0.01, or *** *p* < 0.001) in the figures depending on the significance.

## 3. Results

### 3.1. Clinical Characterization of a Case with Early-Onset STGD1 Reveals Symmetric Disease Development between the Two Eyes

**Figure 1:**
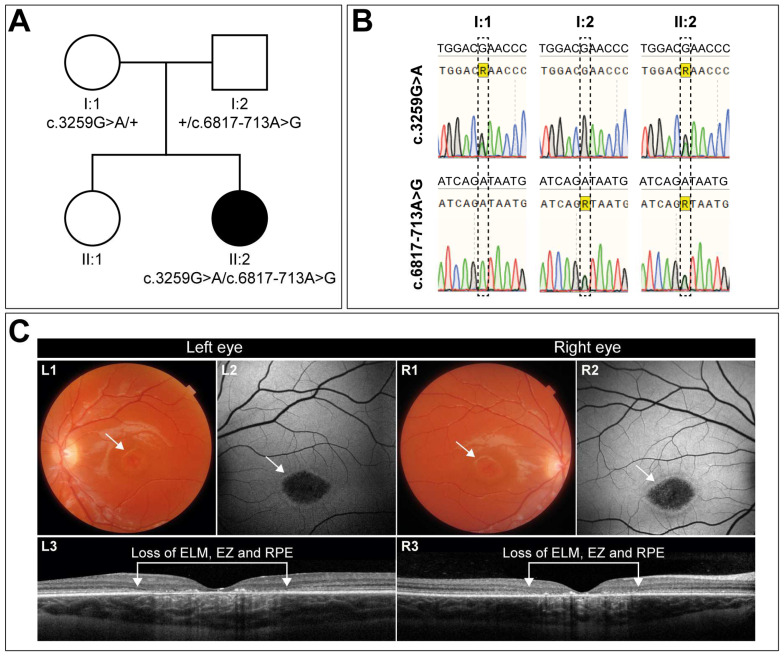
**Mutation analysis and clinical description of the patient and her family.** (**A**) Pedigree showing the segregation of disease. Parents (I:1, I:2) are each heterozygous for one of the two variants. The affected daughter (II:2) is compound heterozygous, harboring both variants. The sibling (II:1) was not analyzed and did not present any symptoms. (**B**) Sanger sequencing traces of the two *ABCA4* regions encompassing the respective variants. (**C**) Imaging of the left and right eye of the patient demonstrates an identical phenotype, with a visual acuity of 0.16 (Snellen decimals). Color fundus photography reveals macular alterations indicated by the white arrows (L1, R1). Central retinal pigment epithelium (RPE) atrophy is evident in both eyes as not well-demarcated hypoautofluorescent areas, encircled by a hyperfluorescent ring, as indicated by the white arrows in the fundus autofluorescence images (L2, R2). Transfoveal OCT scans (L3, R3) show corresponding retinal layer loss of the ellipsoid zone (EZ), external limiting membrane (ELM), and the RPE, along with lipofuscin depositions.

A 10-year-old girl, diagnosed with early-onset STGD1, presented first with complaints of visual decline at the age of 7. The clinical diagnosis was confirmed by the presence of two variants in the *ABCA4* gene: the missense variant c.3259G>A (p.(Glu1087Lys)) in *trans* with the c.6817-713A>G (p.[=,Gln2272_Asp2273ins*11]) change (Figure 1A,B). At the time of diagnosis, her visual acuity had already declined to 0.2 in both eyes. Two years later, her best-corrected visual acuity was 0.16 Snellen decimals for the left and right eye. Slit lamp examination of the exterior and anterior segment was unremarkable.

Her eyes show remarkable symmetry, not only functionally but also structurally (Figure 1C). Color fundus photography (Figure 1C, L1 and R1) and fundus autofluorescence imaging (Figure 1C, L2, and R2) revealed central macular atrophy, with not-well-demarcated borders. Both eyes exhibited a strikingly similar phenotype with a hyperautofluorescent ring surrounding the atrophic area, with comparable sizes of 2.645 mm^2^ for the right and 2.510 mm^2^ for the left eye. This corresponded to an area of retinal layer loss, as observed on OCT scans (Figure 1C, L3, and R3). Flecks were absent in both eyes. If left untreated, the disease is expected to progress further, ultimately leading to severe visual impairment. Given the symmetry between her eyes, this patient is a very good candidate for a one-eye treatment protocol with the other eye serving as the control, offering a unique opportunity to assess therapeutic efficacy.

### 3.2. AONs Can Rescue the Splicing Defect Caused by c.6817-713A>G in Minigene Assays

**Figure 2:**
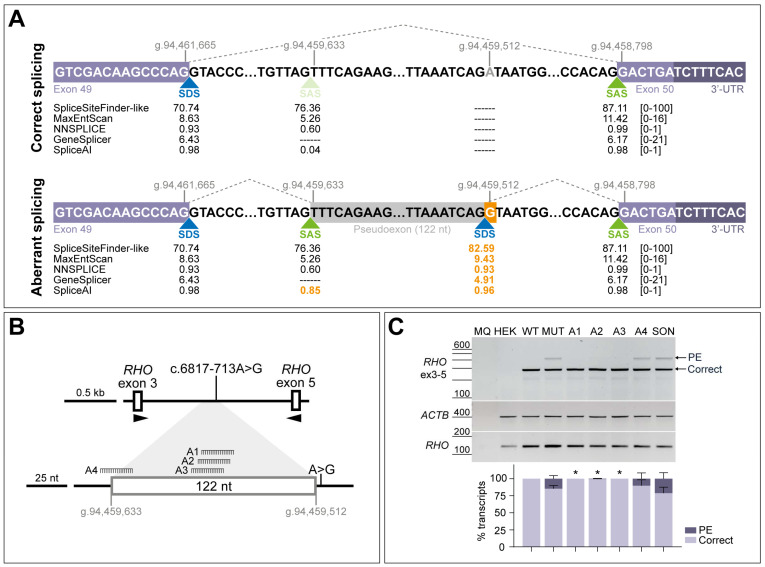
**Schematic representation of the splicing defect caused by variant c.6817-713A>G and initial screening of four antisense oligonucleotides (AONs) in minigene splice assays.** (**A**) Boundaries of the pseudoexon inclusion of 122 nt and flanking exons (GRCh37/hg19 genomic positions). Dashed lines represent the correct splicing process between the canonical splice sites and aberrant splicing between canonical and cryptic splice sites due to the presence of variant c.6817-713A>G (highlighted in orange color). Scores for strength of the different splice donor sites (SDS, in blue color) and splice acceptor sites (SAS, in green color) were predicted with AlamutVisual Plus 1.7.1 software and SpliceAI, indicating that the substitution creates an SDS (scores highlighted in orange color). The score range from each prediction tool is indicated between brackets. (**B**) Depiction of the minigene construct used in splice assays and relative position of the designed AONs (A1, A2, A3, and A4) along the PE sequence. A1-A3 are targeting two high-scored exonic splicing enhancer (ESE) motifs, whereas A4 is targeting the cryptic SAS used for the PE inclusion. (**C**) AON-mediated rescue in minigene-transfected HEK293T cells. Analysis of correct (Correct) and pseudoexon (PE)-including *ABCA4* transcripts by RT-PCR. wild-type (WT) minigene and the respective mutant (MUT) minigene harboring variant c.6817-713A>G were transfected in HEK293T cells. Non-transfected cells were used as endogenous expression control (HEK). The four AONs were then delivered at 0.5 µM, except for the non-treated MUT lane. Scrambled oligonucleotide (SON) delivery at 0.5 µM was used as negative control. Below the representative gel image: semi-quantification analysis graph of the different RT-PCR products is represented, indicating the percentages of the observed *ABCA4* transcripts in each condition. Amplification of β-actin (*ACTB*) gene was used as loading control, and exon 5 of the rhodopsin (*RHO*) gene was used as a minigene transfection control. MQ is used as negative control of all reactions. Data (n = 3) are presented as mean ± SD. Statistical significance is indicated as * *p* < 0.05 using one-way ANOVA test with Dunnett’s multiple comparison analysis, in which non-treated MUT column was the reference condition for correct transcript levels.

The intronic variant c.6817-713A>G was previously described to induce the insertion of a 122-nt PE in minigene assays [9], confirming the prediction of an acceptor gain at position c.6817-835 (already existing as cryptic splice site) and a donor gain at position c.6817-714 (newly created by the nucleotide change) (Figure 2A). Four antisense sequences were designed to rescue the splicing defect; three of the AONs (A1-A3) are targeting two strong SC35 exonic splice enhancer (ESE) motifs within the PE sequence (c.6817-781_c.6817-774 and c.6817-774_c.6817-767), whereas the fourth designed AON (A4) targets the cryptic splice acceptor site at position c.6817-835 (Figure 2B and Figure S1). Splicing correction efficacy of these oligonucleotides was tested in HEK293T cells by using a minigene assay. Transfection of the wild-type minigene showed 100% of the correct transcript, whereas the presence of the variant led to the appearance of the 122-nt PE insertion in the mutant minigene condition, representing 15% of the total transcript (Figure 2C). Upon AON delivery, aberrant splicing was significantly redirected into the formation of correct transcript (Figure S2A), except for A4, which also showed a slight correction but was outperformed by the other oligonucleotides. Transfection with the scrambled oligonucleotide (SON) control seemed to increase the presence of the PE, although this effect is not statistically significant compared to the non-treated condition. Based on these results, all four AONs were tested again in patient-derived fibroblasts to check their effect in a larger genomic context and in a more-relevant molecular environment.

### 3.3. AON-Driven Splicing Correction Is Recapitulated in Patient-Derived Fibroblasts

**Figure 3:**
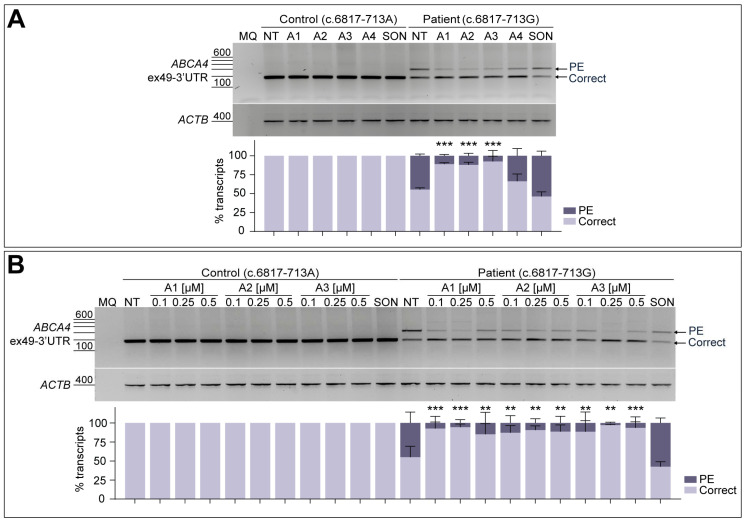
**AON-mediated rescue of aberrant splicing in patient-derived fibroblasts.** Initial testing of the four designed AONs (**A**) and further -dose-response curve of the best-performing AONs (**B**) in control-individual- and patient-derived fibroblasts carrying variant c.6817-713A>G in heterozygosity. Analysis of correct (Correct) and pseudoexon (PE)-including *ABCA4* transcripts by RT-PCR. The four designed AONs (A1–A4) were first tested at 0.5 µM, and increasing concentrations (0.1, 0.25, and 0.5 µM) of the lead candidates (A1–A3) were then delivered in a follow-up assay. Scrambled oligonucleotide (SON) delivery at 0.5 µM was used as negative control. Non-treated (NT) fibroblasts were used as endogenous *ABCA4* expression control. Below the representative gel images, semi-quantification analysis graphs of the different RT-PCR products are represented, indicating the percentages of the observed *ABCA4* transcripts in each condition. Amplification of β-actin (*ACTB*) gene was used as the loading control. MQ is used as the negative control of all reactions. Data (n = 3) are presented as mean ± SD. Statistical significance is indicated as ** *p* < 0.01 and *** *p* < 0.001 using one-way ANOVA test with Dunnett’s multiple comparison analysis, in which non-treated (NT) column was the reference condition for correct transcript levels.

In order to further analyze the splicing modulation capacity of the four potential AON candidates, a fibroblast cell model derived from the STGD1 patient carrying the c.6817-713A>G variant in compound heterozygosity was employed. For these experiments, a control line derived from a healthy individual harboring the *ABCA4* reference sequence was taken along. As observed in Figure 3A, the RT-PCR from exon 49 to the 3′-UTR of *ABCA4* only showed the presence of the correct transcript in case of the control line (Figure S2B). When analyzing the non-treated patient fibroblasts, *ABCA4* transcript levels with the 122-nt PE inclusion increased up to 44.6% of the total amount. For both lines, all four AONs were delivered at a final concentration of 0.5 µM, and none of them had an effect on control conditions. Similarly to the minigene splice assay, A4 was again the least effective among all tested oligonucleotides, despite decreasing the PE inclusion down to 33.7% in patient fibroblasts (Figure 3A). The three AONs targeting the ESEs within the PE sequence (A1, A2, and A3, Figure S1) were able to significantly increase correct splicing, while SON showed no effect on PE inclusion.

The best-performing candidates obtained in this screening were selected for further evaluation at different doses, which were 0.1, 0.25, and 0.5 µM (Figure 3B), excluding A4 from further analyses. In line with the previously described results, none of the delivered AONs or SON to the control line had an effect on normal splicing, in comparison to the respective non-treated condition. As observed in the semi-quantification graph, patient-derived fibroblasts assays presented variability between replicates since some standard deviations can be noticed for the majority of conditions. A strong correlation between the dose and the effect was not detected for any of the AONs, although statistically significant correction was observed for all AON-treated conditions. The SON seemed to have some increased levels of PE inclusion, but this difference was statistically non-significant compared to the non-treated patient cells. Altogether, these results supported further validation of these three potential AON (A1–A3) candidates in patient-derived PPCs.

### 3.4. Patient-Derived Fibroblasts Were Successfully Reprogrammed into Induced Pluripotent Stem Cells (iPSCs)

Patient-derived fibroblasts harboring *ABCA4* variants c.3259G>A and c.6817-713A>G (Figure S3A) were reprogrammed via lentiviral transduction to obtain the corresponding iPSCs, which lost the reprogramming transgene almost completely, as shown by the resulting minimal or no expression from different regions of the delivered lentiviral genome by RT-PCR, while still having it integrated at genomic level (Figure S3B). The newly generated line presented pluripotent characteristics, such as the expression of pluripotency markers, as shown by both qPCR and ICC analyses (Figure S3C,D) and the common iPSCs colony morphology (Figure S3E). This line tested negative for mycoplasma presence in the culture (Figure S3F). In addition, these cells also presented the potency Ito differentiate into all three germ layers, as indicated by the increased expression of the selected markers (Figure S3G). Short tandem repeat (STR) analysis was employed to demonstrate matching patterns between the original fibroblast line that was reprogrammed and the obtained iPSCs. The SNP array results showed no major chromosomal abnormalities (Figure S3H). Common genomic variations often found in the Database of Genomic Variants were detected in cytobands 1p22.1, 2q31.2, 2q37.3, 5q15, 7q31.32, 8q12.1, 11p15.4, 12.p13.31, 16p13.11, 16q24.1, 17p13.3, 17q21.31, 21q11.2, and 22q13.33. An intron deletion and intron duplication in *GRID1* and *HECTD2* genes, respectively, were detected in chromosome 10 (cytobands 10q23.1 and 10q23.32), both reported in the Database of Genomic Variants.

### 3.5. AON Lead Candidates Effectively Rescue Aberrant Splicing in Patient-Derived PPCs

**Figure 4:**
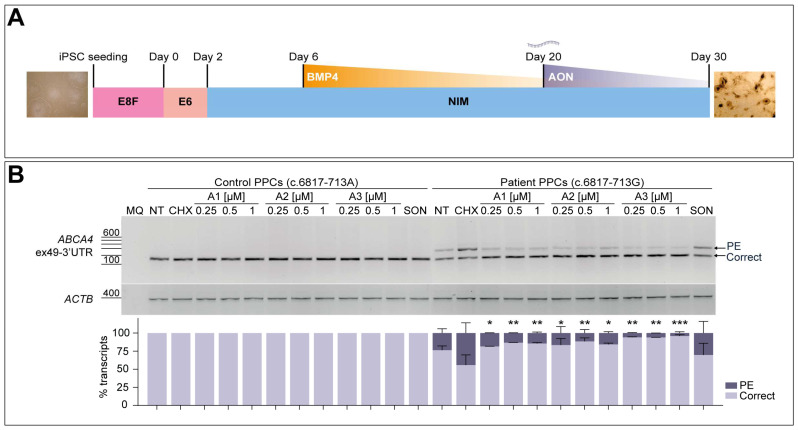
**AON-mediated rescue of aberrant splicing in patient-derived photoreceptor precursor cells (PPCs).** (**A**) Schematic representation of the 30-day protocol used to obtain PPCs from patient-derived induced pluripotent stem cells (iPSCs). Essential 8 Flex (E8F) medium was used for iPSC seeding and at Day 0 was changed to Essential 6 (E6) medium. On Day 2, medium was replaced by neural induction medium (NIM), and later on Day 6, BMP4 pulse was performed. NIM was then refreshed every other day until day 30. (**B**) -Dose-response curve of the best-performing AONs in control-individual- and patient-derived PPCs carrying variant c.6817-713A>G in heterozygosity. Analysis of correct (Correct) and pseudoexon (PE)-including *ABCA4* transcripts via RT-PCR. Increasing concentrations (0.25, 0.5, and 1 µM) of the lead candidates (A1-A3) were delivered on Day 20 of differentiation. PPCs were treated with cycloheximide (CHX) to assess the accumulation of total aberrant transcript or left untreated as endogenous *ABCA4* expression control (NT). Scrambled oligonucleotide (SON) delivery at 1 µM was used as negative control. Semi-quantification analysis of the different RT-PCR products are represented in the graph below the representative gel image. Amplification of β-actin (*ACTB*) gene was used as loading control. MQ is used as negative control of all reactions. Data (n = 2) are presented as mean ± SD. Statistical significance is indicated as * *p* < 0.05, ** *p* < 0.01, and *** *p* < 0.001 using one-way ANOVA test with Dunnett’s multiple comparison analysis, in which the CHX-treated column was the reference condition for correct transcript levels.

The obtained iPSCs from patient-derived fibroblasts were then differentiated into PPCs to further analyze the splicing correction potential of the three best performing oligonucleotides, following a 30-day differentiation protocol as depicted in Figure 4A. In parallel, a control individual iPSC line harboring the wild-type *ABCA4* sequence was employed in the assay. The characterization of these cultures at day 30 via qPCR analysis indicated an increase in *ABCA4* expression in all four differentiations, together with a decreased *OCT4* expression when compared to day 0 iPSC samples (Figure S4). Expression of *PAX6* and *SIX6* neuroretina precursor markers increased in all PPC lines, which was also observed for the photoreceptor progenitor-specific maker *CRX*, showing an early retinal differentiation stage in these cultures. The rod photoreceptor marker *NRL* and the S-cone opsin marker *OPN1SW* presented higher expression at day 30 of differentiation, although the control line showed some variability in the case of *NRL* and *OPN1SW* genes expression. Both lines presented increased expression of the photoreceptor marker *RCVRN*. For all cases, the RPE cell marker *VMD2* confirmed the appearance of these cells in the PPC cultures. Altogether, the characterization of this retina-like cell model suggested the presence of a heterogeneous cell population which also showed high levels of *ABCA4* expression, making it adequate for further AON screening.

The presence of the PE inclusion was clearly observed in the patient-derived PPCs when NMD was inhibited by CHX treatment (Figure 4B). AONs were delivered gymnotically on day 20 at three different concentrations (0.25, 0.5, and 1 µM) based on previous -dose-response curve experiments on the corresponding patient-derived fibroblasts, in this case starting at a slightly higher concentration since no significant rescue was observed at the lowest. Exposure of control-individual-derived PPCs to the three different AONs or the SON at the highest concentration did not produce any effect on the normal splicing process for this particular region, including the CHX-treated condition. The amount of aberrant transcript detected in the patient-derived PPCs was approximately 45% of the total transcript after NMD inhibition, which was significantly reduced to ~4–20% upon delivery of the AONs. These RT-PCR results were confirmed by Sanger sequencing (Figure S2C). Despite the high efficacy measured in this assay, there was no clear correlation of the concentration delivered and the observed effect, since the levels of correct transcript were similar within the dose range in all three cases. Importantly, it can be noticed that no other remarkable splicing events were produced in any of the conditions. The SON shows a slight decrease in the levels of PE-containing transcript, which goes from 45% down to 30%, but these differences are not statistically significant when compared to the CHX-treated patient PPC group. The assessment of correction capacity of the different AONs in patient-derived PPCs indicated how A3 outperformed the other two molecules, even at the lowest concentration. Overall, the screening of four initial AONs in different cell models served to identify the best-performing oligonucleotides to target this 122-nt PE inclusion, narrowing down the number of potential candidates to be assessed in further experiments.

### 3.6. C-Terminal Domain Alteration Affect ABCA4 Protein Levels and Might Be Amenable to AON-Based Correction

**Figure 5:**
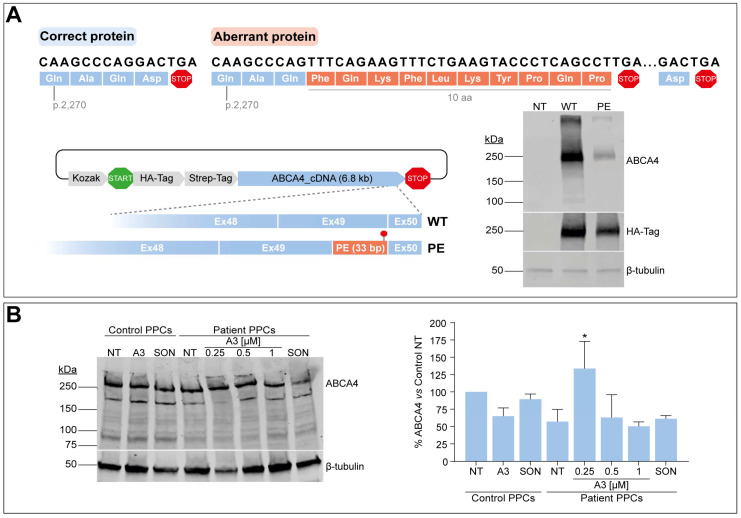
**Assessment of PE effect at the protein level.** (**A**) Open reading frame prediction of the correct and aberrant transcripts by ORF finder. Correct amino acids are depicted in blue color in both correct and aberrant ABCA4 proteins, whereas the extra 10 amino acids added by the PE sequence are depicted in orange color in the aberrant protein. Termination or stop codons are shown in red color. Depicted below, the Gateway p3xHA_CMV/DEST destination vector expressing either the wild-type (WT) *ABCA4* cDNA or presenting the 33-bp insertion between exons 49 and 50 (PE), and the corresponding vector transfection. Western blot analysis (n = 2) of non-transfected (NT) or transfected HEK293T cells with WT or PE *ABCA4* cDNA-expressing vector. HA-Tag was used as transfection control, and β-tubulin was used as a loading control. (**B**) AON-mediated protein rescue in PPCs by western blot analysis. On Day 20 of differentiation, control-individual-derived PPCs were treated with A3 at 1 µM, whereas increasing concentrations (0.25, 0.5, and 1 µM) were delivered to patient-derived PPCs carrying variant c.6817-713A>G in heterozygosity. Both PPC lines were left as non-treated (NT) control, and scrambled oligonucleotide (SON) delivery at 1 µM was used as negative control. β-tubulin was used as a loading control and to normalize ABCA4 levels. Data (n = 2) are presented as mean ± SD in the semi-quantification graph as % of normalized ABCA4 levels to non-treated control. Statistical significance is indicated as * *p* < 0.05 using one-way ANOVA test with Dunnett’s multiple comparison analysis, in which non-treated patient-derived PPCs were the reference condition.

The splicing defect described in this study leads to the addition of 122 nt in between the last two exons of *ABCA4*. As a consequence, the new open reading frame results in the addition of 10 new amino acids followed by a PTC included in the PE sequence, thereby missing the last amino acid of the wild-type protein (Figure 5A). Here, we attempted to investigate the impact of the PE inclusion at the protein level by inserting the first 33 nt from the PE (and including the PTC) between exons 49 and 50 of a vector harboring the entire *ABCA4* cDNA. Once achieved, both vectors expressing either the wild-type or the partial PE-inserted cDNA were transfected into HEK293T, and protein expression was further evaluated by Western blot analysis. As shown in Figure 5A, the presence of the extra 10 amino acids at the end of the protein affected the amount of total ABCA4 protein when compared to the wild-type condition. The corresponding signal from the HA-tag located at the N-term part of the protein also showed a slight decrease in the total protein levels, which might confirm that the extra amino acids are somehow changing the synthesis and/or degradation dynamics of aberrant ABCA4.

As a next step, we questioned whether these alterations at the protein level would also be detectable in our patient-derived PPC model, and if possible, whether this alteration could be rescued upon AON delivery. Control-individual- and patient-derived iPSCs were differentiated into PPCs, which were also characterized via qPCR analysis. In line with the above-described results, all markers showed a similar behavior in both control and patient lines, except for *OPN1SW*, *NRL*, and *VMD2*, whose expression did not increase compared to day 0 of the second replicate (Figure S5A). Despite this, *ABCA4* gene expression increased in all cultures. Similarly to the RNA studies previously described, AON and SON delivery was performed at day 20 of the protocol, but only the lead candidate A3 was taken along in protein assessment experiments. In addition to that, CHX was not used for protein analyses since it inhibits protein translation. ABCA4 signal was detected in all conditions by western blot analysis, with a decrease in total ABCA4 levels in the patient line by >40%, as shown by semi-quantification measurements relative to housekeeping control (Figure 5B). Curiously, several extra bands were detected at different sizes in this protein analysis, which were not present in the HEK293T cells transfected with the construct expressing *ABCA4* cDNA. However, the identity of these bands is not known yet. The control line was treated with the highest amount of A3 and SON (1 µM), whereas the patient line received all three concentrations for A3 (0.25, 0.5, and 1 µM) and the highest for the SON. Again, there was no clear correlation between the dose employed and the detected effect in the case of patient PPCs. Instead, the analysis suggested an increase in ABCA4 protein levels but only at the dose of 0.25 µM compared to the respective non-treated control. In contrast, there is an apparent decrease in ABCA4 levels when the highest concentration is delivered, whereas the SON also reduces these levels. Similarly, A3 and SON treatment at 1 µM had a negative impact on ABCA4 levels in the control PPCs, an event that may be indicating that the protein rescue ability is blocked when higher amounts of the oligonucleotide are used. Altogether, the results at the protein level are too variable to draw any conclusions on whether AON administration increases ABCA4 protein levels, which may be due to the fact that PPCs are not the right model to assess this.

## 4. Discussion

Personalized medicine is gaining recognition within the field of rare diseases, as it might be the most adequate strategy to develop specific treatments that take into account variability in clinical symptoms and biological responses among affected individuals. Currently, improving the clinical design and evaluation of N-of-1 interventions is an important focus of the field [1,2,3,19,37], but preclinical assessment of the potential therapies must be considered as essential in order to standardize aspects that are required prior to trial initiation. For that reason, this study aimed to initiate preclinical testing for the ultrarare deep-intronic *ABCA4* variant c.6817-713A>G, found in one early-onset STGD1 patient, which promotes the insertion of a 122-nt PE between exons 49 and 50 in the final transcript. We developed several AONs that were able to substantially reduce the presence of aberrant transcript in three different in vitro models, including fibroblasts and photoreceptor precursor cells directly derived from the affected individual.

Until now, only a few antisense-based therapies for IRDs managed to enter into clinical testing, which is the case for sepofarsen (*CEP290*-associated IRD), ultevursen (*USH2A*-associated IRD), and QR-1123 (autosomal-dominant retinitis pigmentosa) [38]. AON-based approaches present many advantages and this is why continuous efforts are being made to bring these therapies into the clinical setting for IRDs. Characteristics such as transient alterations at RNA level, long-lasting but non-permanent effect, a relatively easy delivery as naked molecules, and a broad retinal distribution with low risk of undesired systemic effects still qualify AONs as one of the best strategies to target splicing defects. Opting for gene therapies is an alternative mutation-independent approach, but the size of some IRD-associated genes is a serious concern due to cargo capacity limitation of delivery vectors such as adeno-associated viruses [39].

The chemical modifications used for the employed AONs included the phosphorothioate (PS) backbone modification to increase endonucleases resistance and ribose modification 2′-*O*-methoxy-ethyl (2′-MOE) to improve binding affinity to the target RNA, a combination that has been proven to efficaciously rescue PE inclusion defects in *ABCA4* splice assays [12]. In our case, the initial screening in HEK293T cells already gave a hint as to the designed AONs’ efficacy, and in concordance with previous recommendations [24], those molecules targeting exonic splicing enhancer (ESE) motifs are the ones showing the best performance (A1–A3), leaving the sequence targeting the PE splice acceptor site as the least efficacious (Figure 2B). However, it is important to mention that the PE inclusion observed in the mutant minigene was not the major transcript that was produced (Figure 2C), and the defect might be more easily corrected as fewer AON molecules are needed to target a smaller proportion of aberrant pre-mRNA. Apart from this, exons 3 and 5 of *RHO* have strong splice donor and acceptor sites (81.14 and 86.29 according to SpliceSiteFinder-like in Alamut), respectively, which can be preferably recognized by the spliceosome in HEK293T cells and favors the formation of the *RHO* transcript. It may explain, in part, the less prominent PE inclusion compared to other in vitro models.

For that reason, the splicing defect was further validated in different cellular models. The amount of transcripts containing this 122-nt PE inclusion was observed to be more prominent in patient-derived fibroblasts. This demonstrates how essential it is to have a complete genomic context to validate splicing defects, since the endogenous *ABCA4* transcript has a different RNA structure compared to the one produced by the minigene construct. Even though some splicing defects are retina-specific and can only be detected in retina-like models [15,40], the expected 122-nt PE inclusion was also observed in fibroblasts. Like in the minigene assays, A4 was the least efficacious molecule, although a moderate increase in correct transcript was still observed (Figure 3A). Although most characteristics of this sequence fall within the guideline recommendations [23,24], the slightly low GC-content might be reducing the binding strength to the target sequence and, as a consequence, affecting its efficacy to block the splice acceptor site of the PE. The other three tested molecules presented variable rescue efficacy in the performed -dose-response curve on fibroblasts, although similar splicing correction was observed in all conditions (Figure 3B).

In contrast with these results, patient-derived PPCs did not show such a strong PE inclusion in non-treated conditions compared to the corresponding fibroblast line (Figure 4B). Only when NMD was inhibited with CHX, similar aberrant transcripts levels were observed. It is known that NMD plays a crucial role for quality control and regulation of the expressed genes, which can be stimulated by the presence of exon junction complexes downstream a PTC [33]. This might be indicating that, despite being a defect located at the very end of the final mRNA, degradation of the defective transcript may be more dynamic or active depending on the cellular context. However, the total accumulation of aberrant transcript was significantly reduced by all AONs, of which A3 outperformed the other oligonucleotides. Curiously, all three oligonucleotides target a similar region within the PE, and they present a similar length, GC-content, and Tm, so the slightly higher splicing correction driven by A3 is not completely explained by its sequence characteristics. Again, no apparent correlation was detected between the dose and the effect for any of the sequences in PPCs. Altogether, our results at the RNA level reinforce the idea of using several cell models to confirm the splicing events, as there might be noticeable discrepancies between them.

The ten amino acids added by the PE sequence at the C-terminal part of the protein affected the total protein levels. This insertion is predicted to happen at amino acid position 2272, where the very last domains of ABCA4 are localized. Nucleotide-binding domain 2 (NBD2) is followed by the regulatory domain 2 (RD2), and they are part of the cytosolic regions, where their interactions with NBD1 and RD1 help to maintain the overall structure of the NBDs [5]. The distal end of ABCA4 also contains the VFVNFA motif, which is a highly conserved region among ABCA proteins comprising amino acids p.2244–2249, and it is described to be crucial for ABCA4 function. Previous studies have demonstrated that alterations around this motif result in a significant loss of ATPase activity and decreased protein expression [41], which we could also observe in HEK293T cells transfected with the modified *ABCA4* cDNA. In addition, other researchers have demonstrated the important role of VFVNFA motif in its interaction with the NBDs [42]. However, it is also a possibility that the employed antibody is not efficiently binding in the presence of these additional amino acids, since the immunogen used for the manufacturing procedure covers amino acids p.2250 to p.2273 and perhaps decreases the recognition of the epitope. The observed decrease in protein levels detected with the antibody binding to the HA-tag located in the N-terminal end suggests that protein synthesis and/or stability is affected.

On the contrary, the reduction of ABCA4 levels in patient-derived PPCs was not as drastic as observed in HEK293T. The presence of a second allele might be one of the explanations for this observation. The other *ABCA4* mutation carried by the patient is the severe missense variant p.(Glu1087Lys) located within the NBD1 Walker B segment. It is reported to diminish ATPase activity while not affecting protein synthesis [5,43], and consequently, this non-functional protein can still be detected. As a remark, correctly spliced mRNA in analyses should be taken as the contribution of both alleles, while the aberrantly spliced transcript is exclusively being produced by the allele carrying the splice-altering variant. Therefore, it is challenging to determine whether the reduction in protein levels is a result of more NMD activation due the PTC presence or less stability of the aberrant protein for those defective transcripts that managed to be translated with the extra amino acids.

Whereas we could demonstrate a clear rescue at the RNA level in patient-derived PPCs, the results at the protein level were too variable. Despite the apparent positive effect of A3 at the lowest concentration employed (0.25 µM), this was not recapitulated at higher concentrations. Therefore, additional in vitro models are still needed to corroborate the observed effects. It is important to contemplate the premature nature of the retina-like cell model employed in this study, where *ABCA4* expression may not be consistent between lines or differentiations and that can introduce variability when performing semi-quantification analyses. The differentiated PPCs represent a suitable model to filter out the least efficacious sequences for c.6817-713A>G variant, while still presenting the characteristic early photoreceptor markers. Mature photoreceptor cells with fully developed outer segments such as retinal organoids can only be achieved with longer differentiation protocols, which have been previously implemented for the analysis of aberrant *ABCA4* splicing [13,16]. This 3D model can help to elucidate the effect of the different defects at the protein level of the remaining ABCA4. To our knowledge, this is the first time PPCs have been used to assess AON delivery for *ABCA4* at the protein level, which managed to show detectable protein expression and may be still suitable as an early model for treatment assessment. However, the outcome measures at RNA and protein level have to be completed with further studies in, e.g., retinal organoids to demonstrate the efficacy of the best-performing AON found in this research.

Another aspect to consider in future research is the potential toxicity of the designed AONs. The toxicities that are mediated by these molecules can be dependent or independent of the binding of the oligonucleotide, or by the employed chemistry or sequence, which might still lead to immune responses and many other consequences [44,45]. Therefore, it is crucial to have evidence of minimal toxic effects to guarantee the safety of the AON via the performance of the appropriate assays described in previous guidelines [46]. If these aspects cannot be confirmed, further optimization must be carried out in order to obtain a safe lead candidate which, hypothetically, can be used for clinical intervention within the adequate therapeutic window for this specific patient. Once this is confirmed, the strategy for clinical intervention can be designed, but there are a few aspects to consider. One of these is the delivery method to the eye, which is generally performed through intravitreal injection, a well-established and safe method of administration that is frequently used for drug delivery within ophthalmology [47]. As a follow up, the patient must be meticulously monitored for any potential ocular or systemic adverse events, and the therapy’s efficacy should be assessed in stopping disease progression by a comprehensive array of imaging and functional tests. Therefore, sensitive outcome measures should be selected while designing the intervention approach, and in this specific case, atrophy growth appears to be a more suitable marker given that a significant drop in visual acuity has already occurred. Another important aspect is to determine whether a certain treatment is efficacious within a reasonable timeframe. In order to demonstrate efficacy, the approach of treating one eye while using the other eye as a control would be an option for this case, since the patient has remarkable symmetry between both eyes in terms of functionality and structure, a symmetry that makes her a promising candidate for this strategy. Hence, the treatment can be proved efficacious when the treated eye deviates from the disease progression that the other eye shows. Since the patient has already passed the rapid progression phase, a treatment period of 3–5 years of treatment would likely be a realistic period to observe therapeutic efficacy.

Although this patient has already undergone vision loss, further decline is expected. Time is one of the main obstacles to treating any degenerative disease, and the urgency for stopping the progression stimulates the personalization of clinical trials for rare conditions. The rapid approval of other N-of-1 AON-based drugs motivates the development of patient-customized treatments, especially when showing acceptable side-effects but no major safety issues, while improving the quality of life of the affected individual. However, the main downside is that the drug cannot be used to treat a different patient with the same disease, and the limited number of eligible patients also drives other concerns associated with economical, regulatory, and manufacturing issues [22].

## 5. Conclusions

In summary, by implementing the use of different in vitro models directly derived from the affected individual, our work shows the first initiation of preclinical testing of a personalized AON approach to one ultrarare STGD1 case. Further validation work must be conducted to verify minimal safety risks and the potential benefit it can bring to the patient in order to prevent early progression of the disease. Ultimately, our research contributes to customizing AON-based therapy to treat an ultrarare *ABCA4* variant and opens the path for designing an N-of-1 treatment to accelerate clinical intervention for this specific case.

## 6. Patents

R.W.J.C., A.G. and F.P.M.C. are inventors on several filed patents for antisense oligonucleotides (WO2013036105A1, WO2018109011A1, WO2020015959A1, WO2020115106A1, WO2021023863A1), but none of these are used in this manuscript.

## Data Availability

The data supporting the findings of this research are available within the article and supplemental data. Raw data such as unedited blots and gel images are provided; other raw data are available upon reasonable request from corresponding author.

## Supplementary Materials

The following supporting information can be downloaded at https://www.mdpi.com/article/10.3390/cells13070601/s1: Figure S1: Detailed antisense oligonucleotide (AON) design. Figure S2: Validation of the resulting RT-PCR products from AON-mediated rescue experiments; Figure S3: Characterization of the patient-derived iPSC line RMCGENi021-A (021-A); Figure S4: Relative gene expression of pluripotency and retinal markers in PPCs used for RNA analyses; Figure S5: Characterization of the PPCs used for protein analyses; Table S1: Antisense oligonucleotide (AON) sequences and characteristics for the rescue of intronic *ABCA4* variant c.6817-713A>G; Table S2: Primer sequences for site-directed insertion of the *ABCA4* cDNA-containing vector; Table S3: Primer sequences for sequencing validation of the modified *ABCA4* cDNA-containing vector; Table S4: Primer sequences for PPC characterization by qPCR analysis; Table S5: Primer sequences for iPSC characterization; Table S6: Primer sequences for RT-PCR analysis in HEK293T cells, fibroblasts and PPCs rescue studies; Table S7: Semi-quantification analysis of the RT-PCR products from rescue studies in HEK293T cells, fibroblasts and PPCs; Table S8: Semi-quantification analysis of the detected western blot signal in PPC rescue studies.
