# Supplementary material for: Preclinical Development of Antisense Oligonucleotides to Rescue Aberrant Splicing Caused by an Ultrarare ABCA4 Variant in a Child with Early-Onset Stargardt Disease
Source: Cells. 2024 Mar 29;13(7):601. doi: 10.3390/cells13070601 (PMC11011354; doi:10.3390/cells13070601)
Supplement: Supplementary file 1 [file cells-13-00601-s001.zip › cells-2812222-supplementary.pdf]

SUPPLEMENTAL FIGURES

c.6817-713A>G

CGCATGTGCATTCCCAAATTAGGAACAACTCAGATCAATTTCTAATCCTTATTCTTACACTGTTCCAGTTCCCCCA
TATAACTCGTATCTTTGTGTTAGTTTCAGAAGTTTCTGAAGTACCCTCAGCCTTGATGGGGATCCTCGCACCACC
TCAAATCCTGTTCTCAGCCCTAAGAACTGTGTTAGTCATCCTCTTAAGAGGATGTGTGATTTTAAATCAG**G**TAAT
GGGATAAACCACATTTCGTCTAGACTGGTCAGGCCTTTGTCCAGTCCCCTCCTCGCCCACACTACCCCAGCTCCA
CAGCGGGCATTGGTTCAGGAATTCAACCCACAC

SC35 motif

PE inclusion: r.6730_6816ins6817-835_6817–714 (122 nt)

AON1: 5’-GGCUGAGAACAGGAUUUGAG-3’ (target sequence: CTCAAATCCTGTTCTCAGCC)

AON2: 5’-CTGAGAACAGGATTTGAGGT-3’ (target sequence: ACCTCAAATCCTGTTCTCAG)

AON3: 5’-AACAGGATTTGAGGTGGTG-3’ (target sequence: CACCACCTCAAATCCTGTT)

AON4: 5’-CAGAAACTTCTGAAACTAACAC-3’ (target sequence: GTGTTAGTTTCAGAAGTTTCTG)

Figure S1. Detailed antisense oligonucleotide (AON) design. Target sequences of the employed antisense oligonucleotides (underlined), pseudoexon (PE) inclusion (grey), the top scored enhancer splicing motif (green) and variant (yellow) are indicated for the analyzed region (c.6817-934_6817-601).

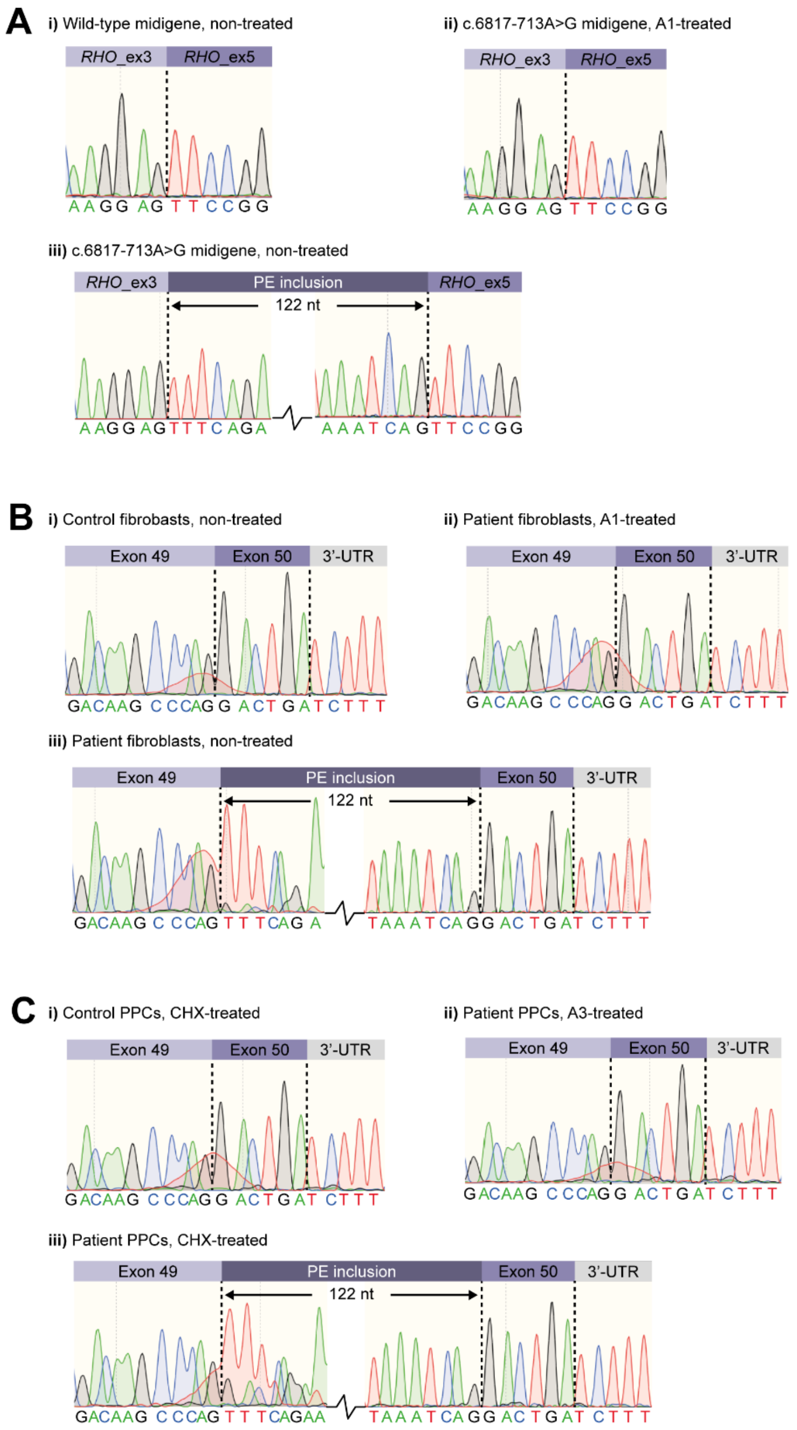

Figure S2. Validation of the resulting RT-PCR products from AON-mediated rescue experiments. Sanger sequencing chromatograms from splicing rescue experiments in HEK293T cells (**A**), fibroblasts (**B**) and PPCs (**C**). For each one, sequences of the correct transcript from non-treated wild-type *ABCA4* minigene, non-treated control fibroblasts or CHX-treated control PPCs conditions (**i**), AON-corrected transcript in mutant *ABCA4* minigene, patient-derived fibroblasts or CHX-treated patient-derived PPCs conditions (**ii**), and PE inclusion transcripts in non-treated mutant *ABCA4* minigene, non-treated patient-derived fibroblasts or CHX-treated patient-derived PPCs conditions (**iii**) are shown.

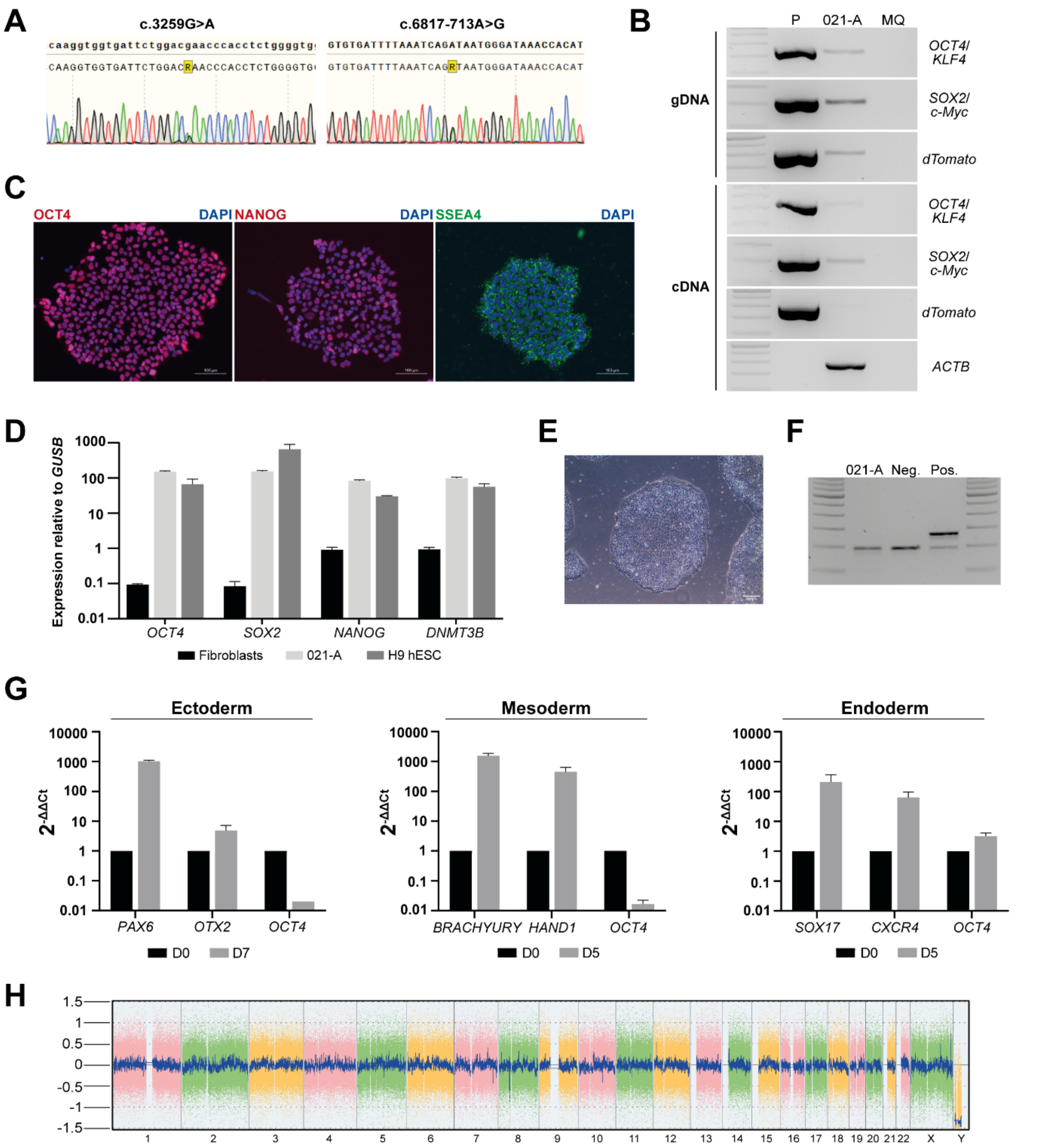

Figure S3. Characterization of the patient-derived iPSC line RMCGENi021-A (021-A). (**A**) Sanger sequencing chromatograms from genomic DNA of the patient line, showing the two *ABCA4* variants that were also present in the reprogrammed cells. (**B**) Vector silencing PCR analysis of the lentiviral vector used for reprogramming, from both genomic DNA (gDNA) and RNA (cDNA) samples. P: plasmid pRRL_PPT_SF_hOct34co_hKlf4co_hSox2co_hmyc_idTomato_pre_FRT, as positive control. MQ is used as negative control of all reactions. (**C**) Pluripotency markers expression in reprogrammed iPSCs by immunocytochemistry (ICC) staining. Scale bar at 100 µm. (**D**) Pluripotency markers expression in reprogrammed iPSCs by qPCR analysis relative to housekeeping gene expression (*GUSB*). The original patient-derived fibroblast sample was used as negative control for pluripotency, whereas the H9 human embryonic stem cell (hESC) line was used as positive control. (**E**) Representative high-contrast image of an isolated iPSC colony by EVOS XL Core Microscope. Scale bar at 200 µm. (**F**) Presence of mycoplasma contamination by PCR from medium collected during iPSC culture. (**G**) Trilineage differentiation capacity of the reprogrammed iPSCs by qPCR analysis. Expression of ectodermal, mesodermal and endodermal markers is shown relative to Day 0 iPSCs. (**H**) Chromosomal aberration analysis of the iPSC line by SNP array.

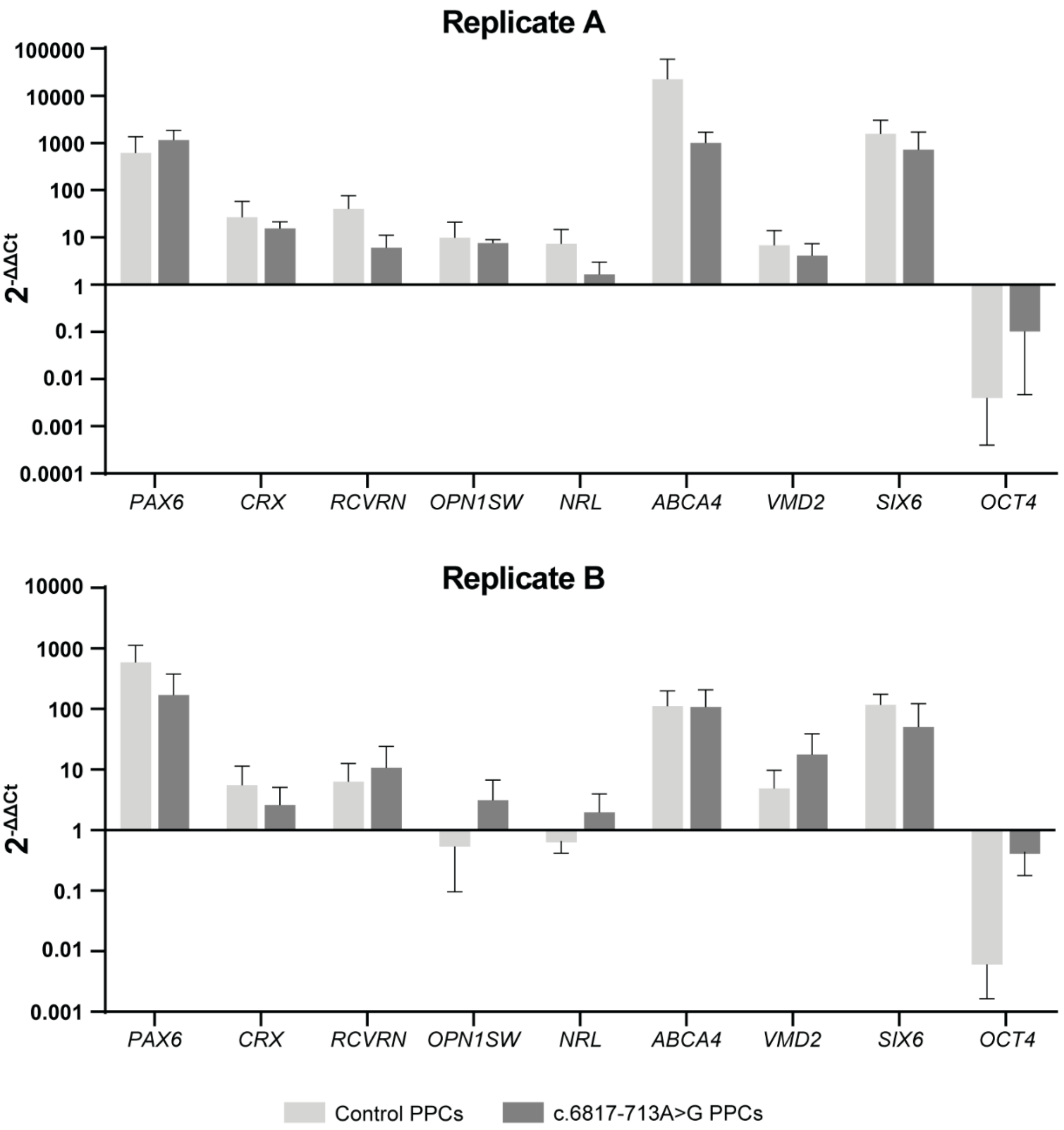

Figure S4. Relative gene expression of pluripotency and retinal markers in PPCs used for RNA analyses. Average gene expression in the control individual and the patient-derived iPSC lines differentiation for replicates A and B. Expression of the retina-specific marker *ABCA4* was increased for both lines and replicates when compared to Day 0 iPSCs. The early neuroretina marker *PAX6* and *SIX6* also presented increased gene expression in all cases. Pluripotency marker *OCT4* expression decreased through differentiation. The RPE cell-specific marker *VMD2* was also increased in all cases. The remaining retinal gene markers *CRX*, *RCV1*, *OPN1SW* and *NRL* also showed increased gene expression, although it was at a lower extent.

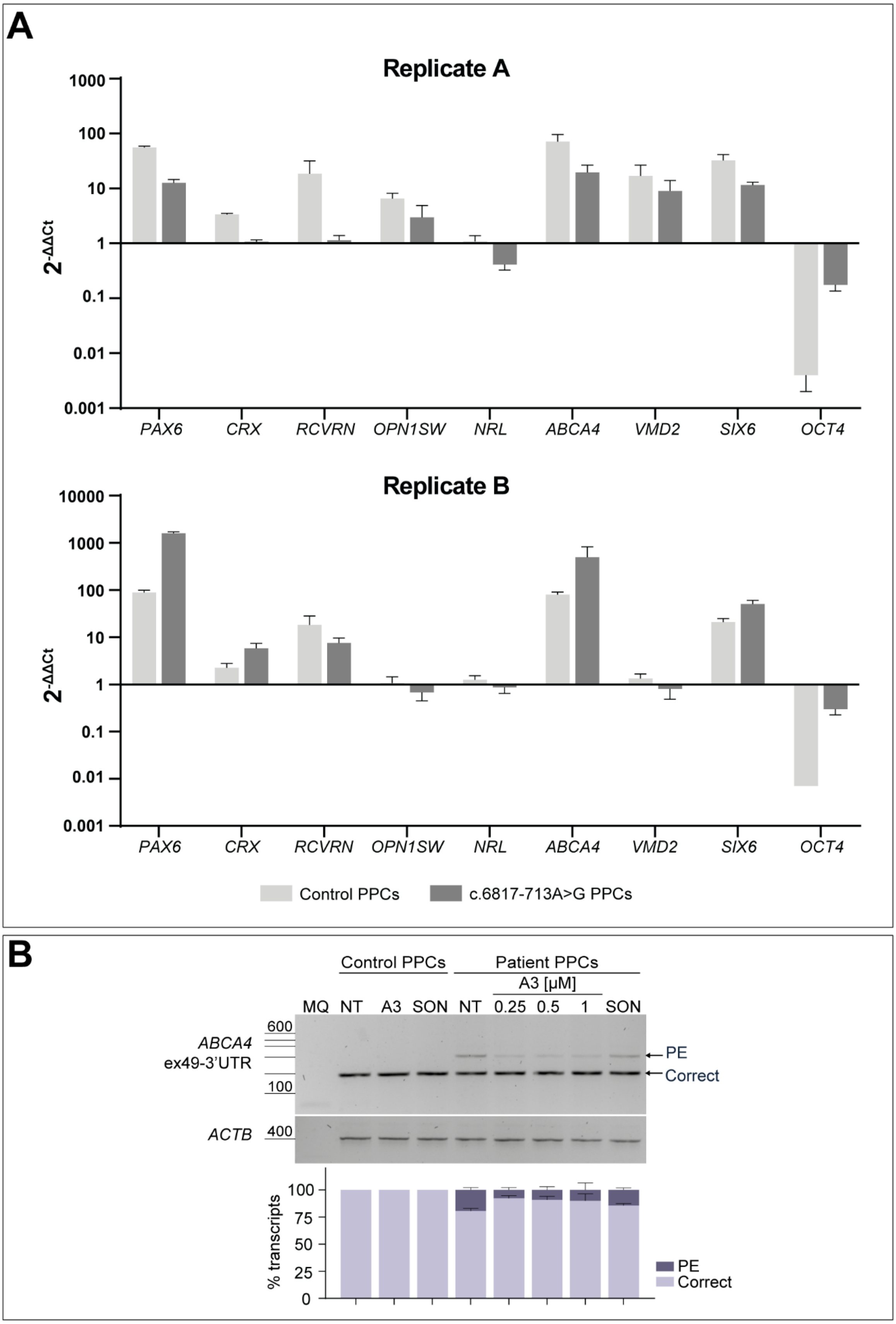

Figure S5. Characterization of the PPCs used for protein analyses. (**A**) Average gene expression in the control individual and the patient-derived iPSC lines differentiation for replicates A and B of protein assays, compared to Day 0 iPSCs. (**B**) A3-mediated rescue at RNA level in control individual and patient-derived PPCs carrying variant c.6817-713A>G in heterozygosity. Analysis of correct (Correct) and pseudoexon (PE)-including *ABCA4* transcripts

by RT-PCR. At Day 20 of differentiation, control individual-derived PPCs were treated with A3 at 1 µM, whereas increasing concentrations (0.25, 0.5 and 1 µM) were delivered to patient-derived PPCs carrying variant c.6817-713A>G in heterozygosity. Both PPC lines were left as non-treated (NT) control, and scrambled oligonucleotide (SON) delivery at 1 µM was used as negative control. Semi-quantification analysis of the different RT-PCR products are represented in the graph below the representative gel image. Amplification of β-actin (*ACTB*) gene was used as loading control. MQ is used as negative control of all reactions. Data (n=2) are presented as mean ± SD. One-way ANOVA test with multiple comparison analysis was employed, in which non-treated (NT) column was the reference condition for correct transcript levels.

SUPPLEMENTAL TABLES

Table S1. Antisense oligonucleotide (AON) sequences and characteristics for the rescue of intronic *ABCA4* variant c.6817-713A>G.

A#	Sequence (5'->3')	Position (cDNA)	Lenght (nt)	% GC	Tm (°C)
A1	GGCUGAGAACAGGAUUUGAG	6817-765_6817-784	20	50,0	51,8
A2	CUGAGAACAGGAUUUGAGGU	6817-767_6817-786	20	45,0	49,7
A3	AACAGGAUUUGAGGUGGUG	6817-772_6817-790	19	47,0	48,9
A4	CAGAAACUUCUGAAACUAACAC	6817-821_6817-842	22	36,0	49,2
SON	CCUCUUACCUCAGUUACAAU	-	20	40,0	47,7

Table S2. Primer sequences for site-directed insertion of the *ABCA4* cDNA-containing vector.

Cloning primer_orientation	Sequence 5'->3'
**PE-insertion_forward**	ttctgaagtaccctcagccttgaGACTAAACCCAGCTTTCTTGTACA
**PE-insertion_reverse**	tacttcagaaacttctgaaaCTGGGCTTGTCGACTGGCTC

Table S3. Primer sequences for sequencing validation of the modified *ABCA4* cDNA-containing vector.

ABCA4-exon_orientation	Sequence 5'->3'
**ex5_forward**	GGAATACGAATAAGGGATATCTTG
**ex6_reverse**	CCACGTTGGCATACAGAGTG
**ex7_forward**	TCTGAGATCTTGGGGAGGAA
**ex11_forward**	CGCCTGGTCAATCAATACCT
**ex13_forward**	GCCTATCTGCAGGACATGGT
**ex15_forward**	GCTGAGCTGAAGAAGGCTGT
**ex19_forward**	TCTTTGAACGTGAGCATCCA
**ex20_forward**	GGGACATTGAAACCAGCCTG
**ex23_forward**	TGCGCAAGATGAAAAACATC
**ex26_forward**	GAAGGTCACGGAGGATTCTG
**ex29_forward**	GCAGTTCACGGTACTTGCAG
**ex31_forward**	AAGACCTGACGGACAGGAAC
**ex36_forward**	CAGTGGATGCTGTGGTTGCCATC
**ex40_forward**	GGGGTGGTGTACTTCCTCCT
**ex45_forward**	GACTGTCTACGCCGACTGC
**ex49_forward**	CAGCAGACTGAAAGTCATGACC

Table S4. Primer sequences for PPC characterization by qPCR analysis.

Gene_primer orientation	Sequence 5'->3'	Transcript size (bp)
***PAX6*_forward**	GCTGCAAAGAAATAGAACATCC	111
***PAX6*_reverse**	TTGGCTGCTAGTCTTTCTCG	
***CRX*_forward**	CCCCAGTGTGGATCTGATG	116
***CRX*_reverse**	CAAACAGTGCCTCCAGCTC	
***RCVRN*_forward**	ACACCAAGTTCTCGGAGGAG	108
***RCVRN*_reverse**	ACTTGGCGTAGATGCTCTGG	
***OPN1SW*_forward**	TTCTTCTCCAAGAGTGCTTGC	97
***OPN1SW*_reverse**	CCTTCCCACACACCATCTTC	
***NRL*_forward**	GGCTCCACACCTTACAGCTC	107
***NRL*_reverse**	AGCCAGTACAGCTCCTCCAG	
***ABCA4*_forward**	CATCCTGTTCCACCACCTCA	113
***ABCA4*_reverse**	CTGTGTCCTCCAACATGGCT	
***VMD2*_forward**	TCAGTGTGGACACCTGTATGC	84
***VMD2*_reverse**	AAGCTGTACACCGCCACAG	
***SIX6*_forward**	CCAGGCAACCGGACTGAC	121
***SIX6*_reverse**	TGTGACAGGACCTGCTGCT	
***OCT4*_forward**	GCAGCAGATCAGCCACATC	119
***OCT4*_reverse**	CCTCTCGTTGTGCATAGTCG	
***GUSB*_forward**	AGAGTGGTGCTGAGGATTGG	80
***GUSB*_reverse**	CCCTCATGCTCTAGCGTGTC	

Table S5. Primer sequences for iPSC characterization.

Gene_primer orientation	Sequence 5'->3'	Transcript size (bp)
***PAX6*_forward**	GCTGCAAAGAAATAGAACATCC	111
***PAX6*_reverse**	TTGGCTGCTAGTCTTTCTCG	
***OTX2*_forward**	AGGAGGTGGCACTGAAAATC	109
***OTX2*_reverse**	TGACCTCCATTCTGCTGTTG	
***BRACHYURY*_forward**	TGCTTCCCTGAGACCCAGTT	121
***BRACHYURY*_reverse**	GATCACTTCTTTCCTTTGCATCAAG	
***HAND1*_forward**	GAACTCAAGAAGGCGGATGG	115
***HAND1*_reverse**	CGGTGCGTCCTTTAATCCTC	
***SOX17*_forward**	GAACGCTTTCATGGTGTGGG	107
***SOX17*_reverse**	CCTTCCACGACTTGCCCAG	
***CXCR4*_forward**	TGGAGGGGATCAGTATATACACT	130
***CXCR4*_reverse**	ATGGTGGGCAGGAAGATTTT	
***OCT4*_forward**	GCAGCAGATCAGCCACATC	119
***OCT4*_reverse**	CCTCTCGTTGTGCATAGTCG	
***SOX2*_forward**	GCTAGTCTCCAAGCGACGAA	144
***SOX2*_reverse**	GCAAGAAGCCTCTCCTTGAA	
***NANOG*_forward**	CTGTGTTCTCTTCCACCCAG	120
***NANOG*_reverse**	TCACCTGTTTGTAGCTGAGG	
***DMNT3*_forward**	TTCCCGGCTACCAGGTCC	88
***DMNT3*_reverse**	CGATGGTAAGGTAAGAGCTGGG	
***GUSB*_forward**	AGAGTGGTGCTGAGGATTGG	80
***GUSB*_reverse**	CCCTCATGCTCTAGCGTGTC	
***OCT4/KLF4*_forward**	ACCCGTGTCCTTTCCTCTG	326
***OCT4/KLF4*_reverse**	TGTTGTTAGGGGCGCCAG	
***SOX2/c-Myc*_forward**	GTCACCAGCAGCTCCCAC	201
***SOX2/c-Myc*_reverse**	GCTCGAATTTCTTCCAGATATCC	
***dTomato*_forward**	GTTCATGTACGGCTCCAAGG	390
***dTomato*_reverse**	TAGTAGTAGCCGGGCAGTTG	
***ACTB*_forward**	ACTGGGACGACATGGAGAAG	384
***ACTB*_reverse**	TCTCAGCTGTGGTGGTGAAG	
***ABCA4*_c.3259G>A_forward**	CACCCTCCACAGCCCCTTAAC	264
***ABCA4*_c.3259G>A_reverse**	AAATGGCAGGTGAGAGAGTG	
***ABCA4*_c.6817-713A>G_forward**	CGCACCACCTCAAATCCTGTTC	331
***ABCA4*_c.6817-713A>G_reverse**	GAGAGATGAGAGCTGGAGTGC	

Table S6. Primer sequences for RT-PCR analysis in HEK293T cells, fibroblasts and PPCs rescue studies**.**

Gene_exon_primer orientation	Sequence 5'->3'	Transcript size (bp)
***RHO*_ex3_forward**	CGGAGGTCAACAACGAGTCT	274
**RHO_ex5_reverse**	AGGTGTAGGGGATGGGAGAC	
***ABCA4*_ex49_forward**	CAGCAGACTGAAAGTCATGACC	167
***ABCA4*_3'UTR_reverse**	GGCCAGTCCATTTGGATGACC	
***ACTB*_ex3_forward**	ACTGGGACGACATGGAGAAG	384
***ACTB*_ex4_reverse**	TCTCAGCTGTGGTGGTGAAG	
***RHO*_ex5_forward**	ATCTGCTGCGGCAAGAAC	140
***RHO*_ex5_reverse**	AGGTGTAGGGGATGGGAGAC	

Table S7. Semi-quantification analysis of the RT-PCR products from rescue studies in HEK293T cells, fibroblasts and PPCs. Correct and aberrant transcripts levels are represented as an averaged percentage (%) of the total transcript for each condition or lane of two independent experiments. SD: standard deviation.

	**Wild-type minigene**		**c.6817-713A>G minigene**											
Condition	NT		NT		A1		A2		A3		A4		SON	
	Mean	SD	Mean	SD	Mean	SD	Mean	SD	Mean	SD	Mean	SD	Mean	SD
% Correct	100,00	0,00	85,43	4,71	100,00	0,00	99,38	0,54	100,00	0,00	89,79	8,54	78,75	8,82
% Aberrant	0,00	0,00	14,57	4,71	0,00	0,00	0,62	0,54	0,00	0,00	10,21	8,54	21,25	8,82

	**Control fibroblasts**											
Condition	NT		A1		A2		A3		A4		SON	
	Mean	SD	Mean	SD	Mean	SD	Mean	SD	Mean	SD	Mean	SD
% Correct	100,00	0,00	100,00	0,00	100,00	0,00	100,00	0,00	100,00	0,00	100,00	0,00
% Aberrant	0,00	0,00	0,00	0,00	0,00	0,00	0,00	0,00	0,00	0,00	0,00	0,00

	**Patient fibroblasts**											
Condition	NT		A1		A2		A3		A4		SON	
	Mean	SD	Mean	SD	Mean	SD	Mean	SD	Mean	SD	Mean	SD
% Correct	55,38	2,39	89,10	1,79	88,07	3,45	92,53	7,11	66,29	9,62	46,13	6,22
% Aberrant	44,62	2,39	10,90	1,79	11,93	3,45	7,47	7,11	33,71	9,62	53,87	6,22

	**Control fibroblasts**																					
Condition	NT		A1_0.1µM		A1_0.25µM		A1_0.5µM		A2_0.1µM		A2_0.25µM		A2_0.5µM		A3_0.1µM		A3_0.25µM		A3_0.5µM		SON_0.5µM	
	Mean	SD	Mean	SD	Mean	SD	Mean	SD	Mean	SD	Mean	SD	Mean	SD	Mean	SD	Mean	SD	Mean	SD	Mean	SD
% Correct	100,00	0,00	100,00	0,00	100,00	0,00	100,00	0,00	100,00	0,00	100,00	0,00	100,00	0,00	100,00	0,00	100,00	0,00	100,00	0,00	100,00	0,00
% Aberrant	0,00	0,00	0,00	0,00	0,00	0,00	0,00	0,00	0,00	0,00	0,00	0,00	0,00	0,00	0,00	0,00	0,00	0,00	0,00	0,00	0,00	0,00

Table S7 (Continued)

	Patient fibroblasts																					
Condition	NT		A1_0.1µM		A1_0.25µM		A1_0.5µM		A2_0.1µM		A2_0.25µM		A2_0.5µM		A3_0.1µM		A3_0.25µM		A3_0.5µM		SON_0.5µM	
	Mean	SD	Mean	SD	Mean	SD	Mean	SD	Mean	SD	Mean	SD	Mean	SD	Mean	SD	Mean	SD	Mean	SD	Mean	SD
% Correct	54,97	14,33	92,57	8,71	94,46	4,24	85,13	13,94	87,05	9,51	90,62	5,59	88,58	8,78	88,44	14,50	97,17	1,04	93,51	7,97	42,53	6,53
% Aberrant	45,03	14,33	7,43	8,71	5,54	4,24	14,87	13,94	12,95	9,51	9,38	5,59	11,42	8,78	11,56	14,50	2,83	1,04	6,49	7,97	57,47	6,53

	Control PPCs																							
Condition	NT		+CHX		A1_0.25µM		A1_0.5µM		A1_1µM		A2_0.25µM		A2_0.5µM		A2_1µM		A3_0.25µM		A3_0.5µM		A3_1µM		SON_1µM	
	Mean	SD	Mean	SD	Mean	SD	Mean	SD	Mean	SD	Mean	SD	Mean	SD	Mean	SD	Mean	SD	Mean	SD	Mean	SD	Mean	SD
% Correct	100,00	0,00	100,00	0,00	100,00	0,00	100,00	0,00	100,00	0,00	100,00	0,00	100,00	0,00	100,00	0,00	100,00	0,00	100,00	0,00	100,00	0,00	100,00	0,00
% Aberrant	0,00	0,00	0,00	0,00	0,00	0,00	0,00	0,00	0,00	0,00	0,00	0,00	0,00	0,00	0,00	0,00	0,00	0,00	0,00	0,00	0,00	0,00	0,00	0,00

	Patient PPCs																							
Condition	NT		+CHX		A1_0.25µM		A1_0.5µM		A1_1µM		A2_0.25µM		A2_0.5µM		A2_1µM		A3_0.25µM		A3_0.5µM		A3_1µM		SON_1µM	
	Mean	SD	Mean	SD	Mean	SD	Mean	SD	Mean	SD	Mean	SD	Mean	SD	Mean	SD	Mean	SD	Mean	SD	Mean	SD	Mean	SD
% Correct	76,31	5,91	55,74	14,19	81,48	0,36	86,60	0,39	85,56	1,42	83,45	9,00	88,48	4,87	84,24	1,90	94,08	0,70	93,98	0,06	95,82	1,85	69,67	16,22
% Aberrant	23,69	5,91	44,26	14,19	18,52	0,36	13,40	0,39	14,44	1,42	16,55	9,00	11,52	4,87	15,76	1,90	5,92	0,70	6,02	0,06	4,18	1,85	30,33	16,22

	Control PPCs						Patient PPCs									
Condition	NT		A3_1µM		SON_1µM		NT		A3_0.25µM		A3_0.5µM		A3_1µM		SON_1µM	
	Mean	SD	Mean	SD	Mean	SD	Mean	SD	Mean	SD	Mean	SD	Mean	SD	Mean	SD
% Correct	100,00	0,00	100,00	0,00	100,00	0,00	80,63	2,33	92,39	2,32	90,94	3,11	90,02	6,46	85,63	1,93
% Aberrant	0,00	0,00	0,00	0,00	0,00	0,00	19,37	2,33	7,61	2,32	9,06	3,11	9,98	6,46	14,37	1,93

Table S8. Semi-quantification analysis of the detected western blot signal in PPC rescue studies. Protein levels are normalized against the housekeeping protein β-tubulin and represented as an averaged value for each condition or lane of two independent experiments. SD: standard deviation.

	**Control PPCs**						**Patient PPCs**									
Condition	NT		A3_1µM		SON_1µM		NT		A3_0.25µM		A3_0.5µM		A3_1µM		SON_1µM	
	Mean	SD	Mean	SD	Mean	SD	Mean	SD	Mean	SD	Mean	SD	Mean	SD	Mean	SD
Area ABCA4 vs β-tubulin	0,87	0,03	0,57	0,13	0,78	0,04	0,50	0,17	1,18	0,39	0,56	0,31	0,44	0,04	0,53	0,02
% ABCA4 vs Control NT	100,00	0,00	64,93	11,78	89,42	7,53	57,05	17,66	133,60	39,40	62,91	32,99	50,29	6,29	61,04	4,60
